# Além do LDL-Colesterol: A Razão Neutrófilos/HDL-Colesterol Avalia o Risco Inflamatório Residual?

**DOI:** 10.36660/abc.20260670

**Published:** 2026-09-25

**Authors:** Henrique Trombini Pinesi, Francisco Akira Malta Cardozo

**Affiliations:** 1 Hospital das Clínicas Faculdade de Medicina Universidade de São Paulo São Paulo SP Brasil Instituto do Coração do Hospital das Clínicas da Faculdade de Medicina da Universidade de São Paulo, São Paulo, SP – Brasil

**Keywords:** Aterosclerose, Inflamação, Infarto do Miocárdio, Neutrófilos, HDL-Colesterol

A redução intensiva do colesterol LDL (LDL-C) transformou a prevenção cardiovascular e reduziu substancialmente a incidência de infarto do miocárdio (IM). No entanto, muitos eventos ainda ocorrem apesar da terapia hipolipemiante otimizada, o que destaca a natureza multifatorial da doença aterosclerótica. Esse risco residual não é uma entidade única: ativação inflamatória, propensão trombótica, disfunção metabólica e lipoproteínas remanescentes aterogênicas atuam em conjunto.^1,2^ Consequentemente, a identificação de biomarcadores capazes de integrar esses processos biológicos complexos tornou-se um objetivo importante na cardiologia preventiva contemporânea.

Neste número dos Arquivos Brasileiros de Cardiologia, Wang et al.^3^ examinam a associação entre a razão neutrófilo-colesterol HDL (RNC) e o IM prevalente em 25.248 adultos da Pesquisa Nacional de Saúde e Nutrição (NHANES), dos quais 1.073 relataram infarto prévio. Após ajuste completo, os participantes no quartil mais alto de RNC apresentaram 49% mais chances de IM do que aqueles no quartil mais baixo (OR 1,49; IC 95% 1,11-2,00), com uma tendência significativa entre os quartis. De forma esclarecedora, a contagem de neutrófilos isoladamente perdeu significância após o ajuste, enquanto o HDL-C manteve uma associação inversa independente.

A justificativa biológica subjacente à RNC é atraente. A aterosclerose é hoje reconhecida como uma doença inflamatória crônica na qual a imunidade inata desempenha um papel fundamental em toda a iniciação, progressão e desestabilização da placa.^1^ Os neutrófilos contribuem para a disfunção endotelial, o estresse oxidativo, a liberação de proteases, a ativação do inflamassoma e a formação de armadilhas extracelulares de neutrófilos, fatores que promovem a vulnerabilidade da placa e complicações trombóticas.^4^ Por outro lado, o HDL exerce múltiplos efeitos protetores que vão muito além do transporte reverso de colesterol, uma vez que as partículas funcionais de HDL possuem propriedades antioxidantes, anti-inflamatórias, antitrombóticas e de proteção endotelial. Em vez de refletir a inflamação ou o metabolismo lipídico isoladamente, a RNC busca quantificar o equilíbrio entre essas forças biológicas opostas, utilizando um único número derivado de exames laboratoriais de rotina.

Talvez o maior valor deste estudo, no entanto, resida não na proposição de mais um biomarcador inflamatório, mas na ilustração de uma transição mais ampla que está ocorrendo na avaliação do risco cardiovascular. Cada vez mais, biomarcadores compostos estão substituindo medições laboratoriais isoladas. A relação neutrófilo-linfócito (RNL), o mais extensivamente estudado desses índices, mostrou que o equilíbrio entre os compartimentos imunológicos carrega informações que nenhuma contagem celular isoladamente transmite. A mesma estrutura conceitual subjaz à relação monócito-HDL (RMH), ao índice sistêmico de inflamação imunológica, ao índice de resposta inflamatória sistêmica e a índices metabólicos como o índice triglicerídeo-glicose: em vez de medir uma única via, eles buscam capturar a interação entre múltiplos sistemas biológicos envolvidos na aterosclerose.^1^ Nesse contexto, a RNL representa mais um exemplo de uma geração emergente de biomarcadores imunometabólicos integrados.

A inflamação é agora um alvo modificável estabelecido, em vez de um epifenômeno. O estudo CANTOS demonstrou que o bloqueio da interleucina-1-beta reduz eventos recorrentes independentemente da redução de lipídios,^5^ e a colchicina reduziu eventos cardiovasculares em contextos coronarianos agudos e crônicos, nos estudos COLCOT e LoDoCo2, respectivamente.^6,7^ Além disso, entre pacientes que recebem terapia com estatinas contemporâneas, a proteína C-reativa de alta sensibilidade prediz eventos e mortalidade com mais força do que o LDL-C.^2^ Qualquer novo marcador inflamatório, portanto, entra em um campo que já possui parâmetros de referência validados.

Alguns aspectos, reconhecidos em parte pelos autores, ajudam a contextualizar esses achados. Primeiro, o delineamento transversal e a dependência de relatos de IM prévio impedem o estabelecimento da temporalidade, e a terapia pós-infarto, a variação de peso e a cessação do tabagismo influenciam a contagem de neutrófilos e o HDL-C. Segundo, um índice composto reflete necessariamente a biologia de seus componentes. O HDL-C é um marcador de risco robusto, embora a randomização mendeliana tenha questionado seu papel causal no IM,^8^ e o fato de o HDL-C ter mantido a significância enquanto a contagem de neutrófilos não, levanta uma questão para trabalhos futuros: quanto do sinal captado pela razão neutrófilo-hemolítico (RNH) é incremental ao HDL-C isoladamente e a marcadores estabelecidos, como a proteína C-reativa de alta sensibilidade? Análises formais de discriminação e reclassificação, que estão além do escopo deste relatório, serão necessárias para responder a essa questão. Terceiro, a RNH aumentou paralelamente à adiposidade, ao tabagismo e ao diabetes em todos os quartis, portanto, é difícil excluir algum viés residual devido à carga cardiometabólica.

Essas considerações definem uma agenda, em vez de diminuir a contribuição. Cohortes prospectivas devem estabelecer se o RNC prediz eventos incidentes, se ele agrega valor aos escores de risco já estabelecidos e — a questão que, em última análise, determina a utilidade clínica — se ele identifica os pacientes que se beneficiam da terapia anti-inflamatória. A dimensão brasileira também merece destaque: nossas diretrizes já recomendam refinar a estratificação além do LDL-C em indivíduos de risco intermediário,^9^ e um índice derivado de exames realizados rotineiramente em qualquer unidade de atenção primária seria interessante em um sistema com recursos limitados.

O interesse do RNC reside, em última análise, na direção que ele aponta: uma mudança de variáveis laboratoriais isoladas para índices imunometabólicos integrados que capturam a interação entre inflamação, imunidade e metabolismo lipídico. Wang et al.^3^ devem ser parabenizados por uma análise cuidadosa e apropriadamente cautelosa que impulsiona essa agenda.
