# Supplementary material for: Além do LDL-Colesterol: A Razão Neutrófilos/HDL-Colesterol Avalia o Risco Inflamatório Residual?
Source: Arq Bras Cardiol. 2026 Sep 25;123(8):e20260670. [Article in Portuguese] doi: 10.36660/abc.20260670 (PMC13668376; doi:10.36660/abc.20260670)
Supplement: Translation

# Translation: Beyond LDL-Cholesterol: Can the Neutrophil-to-HDL-Cholesterol Ratio Capture Residual Inflammatory Risk?

**DOI:** 10.36660/abc.20260670i

**Authors:** Henrique Trombini Pinesi, Francisco Akira Malta Cardozo

**Affiliations:** 1 Brazil Instituto do Coração do Hospital das Clínicas da Faculdade de Medicina da Universidade de São Paulo, São Paulo, SP – Brazil

**Keywords:** Atherosclerosis, Inflammation, Myocardial Infarction, Neutrophils, HDL Cholesterol

Intensive low-density lipoprotein cholesterol (LDL-C) lowering has transformed cardiovascular prevention and substantially reduced the incidence of myocardial infarction (MI). Yet many events still occur despite optimal lipid-lowering therapy, highlighting the multifactorial nature of atherosclerotic disease. This residual risk is not a single entity: inflammatory activation, thrombotic propensity, metabolic dysfunction, and atherogenic remnant lipoproteins all act in concert.^1,2^ Consequently, identifying biomarkers capable of integrating these complex biological processes has become an important goal in contemporary preventive cardiology.

In this issue of the *Arquivos Brasileiros de Cardiologia*, Wang et al.^3^ examine the association between the neutrophil-to-high-density lipoprotein cholesterol ratio (NHR) and prevalent MI in 25,248 adults from the National Health and Nutrition Examination Survey (NHANES), of whom 1,073 reported a previous infarction. After full adjustment, participants in the highest NHR quartile had 49% higher odds of MI than those in the lowest (OR 1.49; 95% CI 1.11-2.00), with a significant trend across quartiles. Instructively, neutrophil count alone lost significance after adjustment, whereas HDL-C retained an independent inverse association.

The biological rationale underlying NHR is appealing. Atherosclerosis is now recognized as a chronic inflammatory disease in which innate immunity plays a pivotal role throughout plaque initiation, progression, and destabilization.^1^ Neutrophils contribute to endothelial dysfunction, oxidative stress, protease release, inflammasome activation, and neutrophil extracellular trap formation, all of which promote plaque vulnerability and thrombotic complications.^4^ Conversely, HDL exerts multiple protective effects extending far beyond reverse cholesterol transport, since functional HDL particles possess antioxidant, anti-inflammatory, antithrombotic, and endothelial-protective properties. Rather than reflecting either inflammation or lipid metabolism in isolation, NHR attempts to quantify the balance between these opposing biological forces, using a single number derived from routine laboratory tests.

Perhaps the greatest value of this study, however, lies not in proposing another inflammatory biomarker, but in illustrating a broader transition occurring in cardiovascular risk assessment. Increasingly, composite biomarkers are replacing isolated laboratory measurements. The neutrophil-to-lymphocyte ratio, the most extensively studied of these indices, showed that the balance between immune compartments carries information that neither cell count conveys alone. The same conceptual framework underlies the monocyte-to-HDL ratio, the systemic immune-inflammation index, the systemic inflammation response index, and metabolic indices such as the triglyceride-glucose index: rather than measuring a single pathway, they attempt to capture the interaction between multiple biological systems involved in atherosclerosis.^1^ In this context, NHR represents another example of an emerging generation of integrated immunometabolic biomarkers.

Inflammation is now an established modifiable target rather than an epiphenomenon. CANTOS showed that interleukin-1-beta blockade reduces recurrent events independently of lipid lowering,^5^ and colchicine reduced cardiovascular events in the acute and the chronic coronary settings, in COLCOT and LoDoCo2 respectively.^6,7^ Moreover, among patients receiving contemporary statin therapy, high-sensitivity C-reactive protein predicts events and mortality more strongly than LDL-C.^2^ Any new inflammatory marker therefore enters a field that already has validated benchmarks.

Some aspects, acknowledged in part by the authors, help place these findings in perspective. First, the cross-sectional design and the reliance on self-reported prevalent MI mean that temporality cannot be established, and post-infarction therapy, weight change and smoking cessation all influence neutrophil count and HDL-C. Second, a composite index necessarily reflects the biology of its components. HDL-C is a robust risk marker, although Mendelian randomization has questioned its causal role in MI,^8^ and the fact that HDL-C retained significance while neutrophil count did not raises a question for future work: how much of the signal captured by NHR is incremental to HDL-C alone, and to established markers such as high-sensitivity C-reactive protein? Formal discrimination and reclassification analyses, beyond the scope of the present report, will be needed to answer it. Third, NHR rose in parallel with adiposity, smoking and diabetes across quartiles, so some residual confounding by cardiometabolic burden is difficult to exclude.

These considerations define an agenda rather than diminish the contribution. Prospective cohorts should establish whether NHR predicts incident events, whether it adds to established risk scores, and - the question that ultimately determines clinical utility - whether it identifies the patients who benefit from anti-inflammatory therapy. The Brazilian dimension also deserves emphasis: our guidelines already recommend refining stratification beyond LDL-C in intermediate-risk individuals,^9^ and an index derived from tests performed routinely in any primary care unit would be attractive in a resource-constrained system.

The interest of NHR ultimately lies in the direction it points to: a shift from isolated laboratory variables toward integrated immunometabolic indices that capture the interplay between inflammation, immunity and lipid metabolism. Wang et al.^3^ should be congratulated for a careful and appropriately cautious analysis that advances that agenda.
